# SPEM dysfunction and general schizotypy as measured by the SSQ: a controlled study

**DOI:** 10.1186/1471-2377-9-27

**Published:** 2009-06-29

**Authors:** Dirk van Kampen, Jan Berend Deijen

**Affiliations:** 1 Department of Clinical Psychology, VU University, Van der Boechorststraat 1, 1081 BT Amsterdam, The Netherlands; 2 Department of Clinical Neuropsychology, VU University, Van der Boechorststraat 1, 1081 BT Amsterdam, The Netherlands

## Abstract

**Background:**

SPEM dysfunction is a well-known phenomenon in schizophrenia. The principal aim of the present study was to examine whether SPEM dysfunction is already observable in subjects scoring high on a specific measure of schizotypy (SSQ General Schizotypy) that was selected because of its intimate relationship with schizophrenic prodromal unfolding.

**Methods:**

Applying ANOVAs, we determined the relationship of subjects' scores on SSQ General Schizotypy and eye movements elicited by targets of different speed. We also examined whether there exists an association between our schizotypy measure and pupil size.

**Results:**

We found more SPEM dysfunction in subjects scoring high on SSQ General Schizotypy than in subjects scoring average on that factor, irrespective of the speed of the target. No relationship was found between baseline pupil size and General Schizotypy.

**Conclusion:**

The present study provides additional evidence that SPEM dysfunction is associated with schizotypic features that precede the onset of schizophrenia and is already observable in general population subjects that show these features.

## Background

One century ago, Diefendorf and Dodge first described a phenomenon in schizophrenic patients that is presently known as smooth pursuit eye movement (SPEM) dysfunction [1]. Rediscovered in the early 1970s by Holzman et al. [2], this phenomenon, reflecting an impairment in the ability to visually track a smoothly moving target, was not only observed in 60–80% of patients with schizophrenia [3,4], but also in approximately half of their biological relatives [5,6], and in monozygotic twins who were discordant for schizophrenia [7]. Other studies indicated that SPEM impairments occur in abnormally high proportions in patients suffering from schizotypal personality disorder or SPD [8,9]; these findings are understandable as SPD is known to be genetically related to schizophrenia [10,11]. Finally, eye tracking impairments have also been observed in general population subjects with elevated scores on questionnaire measures of schizotypy [12]. Given the above-mentioned findings, SPEM dysfunction has been typically regarded as a neurophysiological marker of the genetic vulnerability to develop schizophrenia [4,13]. According to Holzman et al. [14], eye tracking disturbance and schizophrenia are both expressions of a single underlying trait that is transmitted as an autosomal dominant gene. Additional evidence for a single major gene accounting for much of the variance in eye tracking performance was obtained by Grove et al. [15]. However, at present no firm conclusions can be drawn as several association and linkage studies suggest the influence of multiple genes. Still, in the last ten years, no eye movement dysfunction related genes for schizophrenia have been definitely identified [16].

In addition to the general relationship of SPEM abnormalities with schizophrenia and SPD, the association between deviant eye tracking and specific symptoms of these disorders has been studied. Most studies indicate a relationship with negative symptoms of schizophrenia [17-21] and SPD [9,22-24], but a relationship with positive symptoms and symptoms related to Liddle's disorganization syndrome [25] have also been reported [5,24,26,27]. Similarly, SPEM dysfunction has been found in general population subjects with elevated scores on either positive or negative measures of schizotypy [28-30]. The close relationship of SPEM dysfunction with negative symptoms agrees with the notion that the genetic predisposition to schizophrenia expresses itself predominantly in negative symptoms [31-36]. However, positive symptoms may also reflect the genetic basis of schizophrenia. In Kendler et al's study, for instance, nearly all schizotypal factors, including positive and negative schizotypy, discriminated relatives of schizophrenic probands from relatives of controls [33]. Even, elevated rates of only positive schizotypy have been found in biological relatives of schizophrenics [37,38]. To explain these conflicting results, the finding is of interest that individuals with elevated scores on negative and positive schizotypy appear at particularly high risk for development of schizophrenia, whereas those with deviant scores on only scales for positive schizotypy may also develop other types of psychotic disorder [39]. As negative symptoms precede the emergence of positive symptoms in schizophrenia [40-42], we may infer that the genetic predisposition to schizophrenia manifests itself first in negative symptoms. Subsequently, psychotic-like and psychotic symptoms, may develop which reflect schizophrenia's pathophysiology and genetics to a lesser degree and only in an indirect way [43].

Concerning the measurement of SPEM dysfunction, some studies have focused on global ocular motor measures, whereas others evaluated saccade frequency and pursuit gain [22]. Formerly, these latter measures have been reported to be less sensitive than global assessments [44,45]. Indeed, in a recent meta-analytic review of smooth pursuit in schizophrenia, global measures, such as the RMSE, were found to yield larger effect sizes than specific measures [46]. The discriminative power of the RMSE may be the product of the inability of the subject to pursuit the target at the same speed as well as to suppress the saccade system leading to intrusive saccades. Thus, although the RMSE has excellent test-retest reliability and may be useful in screening situations, it is not useful for clarifying specific deficits [47]. However, as our aim was not to reveal specific deficits but to relate a robust measure of SPEM dysfunction with schizotypy, we choose to measure the RMSE.

In the present study schizotypy was assessed by the general factor of Van Kampen's *Schizotypic Syndrome Questionnaire *(SSQ) [48]. The reason for this choice was twofold. First, the 12 symptom scales of the SSQ were selected on basis of previous studies [40,42,49] to describe the main causal pathways in the process of schizophrenic prodromal unfolding [43,50]. Between the selected symptoms a detailed network of causal relationships was postulated against the background of the observation that negative symptoms precede positive ones. In addition, the 12 SSQ scales offer a definition of schizotypy (and SPD) with particular relevance for the study of variables that relate to both schizotypy and schizophrenia. Second, as the SSQ higher-order factors Negative Schizotypy, Asocial Schizotypy, and Positive Schizotypy appear to be interrelated [48], the general SSQ factor can be considered to accurately denote what schizotypy is really like. As also negative and positive schizotypy factors have been found associated with SPEM dysfunction, we decided to investigate the relationship between schizotypy and SPEM impairments by using the general SSQ factor. However, it remains of interest to see that, although all SSQ scales load the general factor, the highest saturations were obtained for those scales that define the Negative Schizotypy factor of the SSQ [48].

In addition to SPEM performance, schizophrenia has been associated with a baseline pupil size that correlated positively with symptom severity as measured by the *Brief Psychiatric Rating Scale *and the *Hamilton Rating Scale for Depression*, although not with several scales for the measurement of negative and/or positive symptoms [51].

In the present study, the relationship between subjects' scores on the SSQ and eye movements elicited by targets of different speed, was established. It was expected that higher scores on the General Schizotypy factor would be associated with more apparent SPEM dysfunction, the magnitude of which being possibly dependent on target speed. In addition, we examined whether there exists a relationship between General Schizotypy and pupil size. What is novel in this study compared to previous ones is the use of a very particular measure of schizotypy for mainly theoretical reasons. As such, the present investigation may also add to the validation of the SSQ model with its emphasis on a general factor.

## Methods

### Subjects

The present study forms part of a larger study on the validity of the SSQ [48]. A number of 495 subjects (290 females, 205 males), drawn from patient files of four general practitioners in Amsterdam, were invited by letter to fill in the SSQ and the *Four-Dimensional Personality Test *(4DPT) [52] that were enclosed in the mail. Subjects were aged between 20 and 50 years. On the closing date, after two weeks, 140 SSQ and 4DPT forms had been returned by 48 male and 92 female subjects. Subjects had a mean age of 35.97 years (SD = 7.03). Noting that the scores on the 12 SSQ scales appeared to be somewhat higher than those obtained in a more representative sample of 557 subjects (all inhabitants of Haarlem and Gouda [43]), the General Schizotypy scores in the present group were estimated on the basis of a regression equation derived in the last-mentioned sample. After discarding 9 subjects with insufficient data to estimate the scores on the first unrotated component, three subgroups were formed; 1) a group of subjects with General Schizotypy scores above 1 SD (n = 33), 2) a group of subjects with General Schizotypy scores between -1 and +1 SD (n = 85), and 3) a group of subjects with General Schizotypy scores less than -1 SD (n = 13). As it is clear from these numbers that the distribution of scores is positively skewed (0.82), the use of SD in dividing the sample is somewhat problematic. Nevertheless, we decided to maintain our criteria. All 33 high scoring subjects, as well as 33 subjects randomly drawn from the group with scores between -1 and +1 SD, were invited to participate in a SPEM experiment. As at most 13 subjects of the low scoring group could be expected to participate, this group was not invited.

A number of 25 subjects from the high scoring group and 22 from the group with 'average' scores were willing to participate. These 47 subjects had a mean age of 37.30 years (SD = 7.33). There were no differences between the two subgroups regarding sex and age. Due to the occurrence of eye blinks and the non-registration of pupil sizes less than 2 mm, dependable SPEM data could be obtained for 40 subjects: 19 individuals with elevated scores on General Schizotypy and 21 persons with 'average' scores. As three individuals – one from the high scoring and two from the average scoring group – had outlying RMSE scores in at least one of the three speed conditions mentioned below, the final sample consisted of 37 subjects. The 18 subjects (9 females, 9 males) in the high schizotypy group had a mean age of 37.44 years (SD = 6.64), and the 19 subjects (10 females, 9 males) with average schizotypy scores had a mean age of 36.11 years SD = of 6.83). The two subsamples were not significantly different in age and gender. Ethical approval for the study was obtained from the review committee of the Faculty and all participants gave informed consent.

### Procedure

#### SSQ and 4DPT measures

The SSQ [48] consists of 12 scales for the measurement of the schizotypic symptoms Social Anxiety (SAN), Active Isolation (AIS), Affective Flattening (AFF), Apathy (APA), Alienation (ALN), Living in a Fantasy World (FTW), Egocentrism (EGC), Suspicion (SUS), Hostility (HOS), Cognitive Derailment (CDR), Perceptual Disturbances (PER), and Delusional Thinking (DET). As already indicated, the instrument breaks down into three correlated factors – Negative Schizotypy, Asocial Schizotypy, and Positive Schizotypy. The general factor of the SSQ has loadings from all scales, but particularly from scales that measure negative symptoms. The SSQ demonstrates adequate reliability, with Cronbach's alpha coefficients ranging from 0.77 to 0.91 in a sample of 771 general population subjects. Meaningful correlations, attesting the construct validity of the SSQ, were obtained by relating the 12 scales to several other instruments, among which the *Schizotypal Personality Questionnaire *[53] and the *Dissociative Experiences Scale-II *[54]. The items of the SSQ, which are presented in the form of statements pertaining to behaviour and feelings, must be answered on a four-point scale, indicating the degree to which each statement applies to the subject. The higher the scores on the 12 SSQ scales, the higher the schizotypy.

The 4DPT or *Four-Dimensional Personality Test *[52] was used to evaluate the validity of the SSQ. It was constructed in an attempt to improve Eysenck's PEN model by showing that his P or Psychoticism construct [55], as measured by the *Eysenck Personality Questionnaire*, actually refers to two distinct personality factors, S or Insensitivity and G or Orderliness. In contrast, the factors E (Extraversion) and N (Neuroticism) appeared to be 'non-problematic' and could be retained. The 4DPT scales for S, E, N and G are quite reliable and correlate with four of the five Big Five dimensions. As it is known that the schizoid, schizotypal or pre-schizophrenic personality is characterized by features resembling high S and N positions, and a low position on E [56-58], we expected the SSQ factor General Schizotypy to be positively related with the 4DPT dimensions S and N, negatively with E, and unrelated with G.

#### Oculomotor assessment

Eye movements were recorded by the Whittaker 1998-S Eye View Monitor and TV Pupillometer System, an infrared video-pupillometric system (Whittaker Corporation, Waltham, Massachusetts) [59]. This system generates video images and values of horizontal and vertical positions. These positions are based on the measurement of pupil size that is sampled with a frequency of 50 Hz. A sampling frequency of 50 Hz is generally considered not to be suited for measuring fast eye movements. However, recently it was shown that video eye trackers sampling at 50 Hz are appropriate for detecting the clinical relevant saccade peak velocities [60]. Thus, even with our low temporal resolution of 50 Hz it is possible to measure pursuit gain and saccades during pursuit. However, as it was our purpose to relate a robust measure of SPEM dysfunction with schizotypy we choose to measure the RMSE. As a sample frequency of 50 Hz may be suited to measure fast eye movements, it can certainly considered appropriate for the assessment of the RMSE.

Subjects were tested individually in a sound attenuated room. They were instructed by the experimenter though an intercom system. After a 7-min dark-adaptation period, the testing procedure started. Each subject sat at a distance of 57 cm in front of a monitor, the head restrained using a chin rest. An infrared light camera, fastened in an ophthalmologic framework, was positioned at the height of the subject's left eye limbus, registering left eye movements and pupil size. Because of practical restrictions of the pupillometric system, the measurement was unilateral (left eye) and recordings could not be evaluated if the pupil size was less than 2 mm. The pupil diameter resolution of the system was 1 part in 512 full scale. This size was measured in mV, which value was divided by 50 to get the pupil's diameter. Furthermore, no registrations were retained in which no tracking occurred. The target that consisted of a small square of white light that moved horizontally on a black background screen was originally positioned at the left site seen from the middle of the monitor screen. The subject was instructed to follow the target as carefully as possible and to try not to blink. After one second and a half, the target began to move to the right. The angle between the eye and the turning point was 15 degrees. In line with the study of Smyrnis et al. in which RMSE was found to increase with increasing target speed (10, 20 and 30 deg/s), we included targets with different speed [47].

#### Data analysis

The eye movement data were analysed under three conditions of target speed. The lowest speed (L, 5°/s) was presented between 92,500 and 184,000 ms, the medium speed (M, 10°/s) between 189,000 and 238,000 ms, and the highest speed (H, 20°/s) between 248,000 and 284,000 ms. Eye movements were analysed only when the target was moving but not during the 1 sec time the target stand still and they were not taken into account in case of too many eye blinks. As already indicated, we choose a rather global measure of SPEM (dys)function, the root mean square error score (RMSE) for eye vs. target position. For each of the three conditions of target speed, an RMSE score was calculated. In the analysis of the relationship between General Schizotypy and SPEM dysfunction, we first applied ANOVA with group (low vs. high General Schizotypy) and target speed condition as repeated measurements factor on RMSE scores. As three individuals turned out to have outlying scores on RMSE in at least one of the three conditions of target speed, 19 average scoring subjects on General Schizotypy were actually compared with 18 high scoring subjects. Secondly, we applied separate ANOVAs with group as independent factor for each of the target speed conditions. Similar analyses were performed for pupil diameter. As for all target speeds more SPEM deviations were expected to occur among individuals with higher scores on General Schizotypy, the statistical tests with respect to the SPEM data were one-tailed. As we expected no relationship between pupil size and General Schizotypy, this relation was analysed by two-tailed tests. Data were analysed using the SPSS version 14 software package (SPSS inc., Chicago, USA).

## Results

As expected the two subgroups scored significantly different on the first unrotated SSQ factor (*T *(35) = -10.88, *p *< 0.0005, *d *= 3.5). The means of the estimated scores on General Schizotypy is 1.80 (± 0.47) for the subgroup with scores above 1 SD (n = 18) and 0.06 (± 0.50) for the subgroup with scores between -1 and +1 SD (n = 19). In contrast to other studies defining the high schizotypy group as scoring > 2 SD and the average schizotypy group as scoring +/- 0.5 SD [28-30], the two groups in our study were less clearly separated. However, the highly significant effect and large effect size *d *indicates that our subgroups differ substantially in schizotypy score. Also in line with our expectations *t*-tests indicated that the high schizotypy group scored significantly higher on the 4DPT dimensions S and N and lower on E, whereas no differences were observed on 4DPT-G (Mean Insensitivity, low schizotypy group: S = 4.97 ± 3.46; high schizotypy group: S = 7.39 ± 4.21, *p *= 0.047; Mean Neuroticism, low schizotypy group: N = 5.16 ± 4.07; high schizotypy group: N = 11.47 ± 2.85, *p *< 0.0005; Mean Extraversion, low schizotypy group: E = 10.0 ± 4.17; high schizotypy group: E = 6.65 ± 4.57, *p *= 0.028). These findings are additional support of the validity of the SSQ measure.

**Figure 1:**
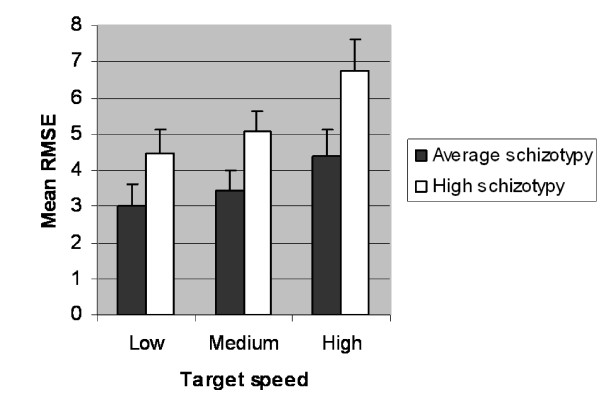
**Means (M) ± Standard Errors (SE) of Root Mean Square Error (RMSE) score for low, medium or high target speed in groups scoring average or high on SSQ-General Schizotypy**.

ANOVA with Group as independent factor and Target speed as repeated measurements factor indicated that the high schizotypy group had significantly higher RMSE scores than the average scoring group (*F *(1,35) = 5,68, *p *= 0.023, *η*^2 ^= 0.14). In addition, no significant interaction between Group and Target speed was found (Figure 1). Univariate ANOVAs with Group as independent factor indicated that for each target speed the high schizotypy group had significantly higher RMSE scores than the average schizotypy group (Low speed: *F*(1,35) = 5.15, *p *= 0.015, *η*^2 ^= 0.13; Medium speed: *F*(1,35) = 3.85, *p *= 0.029, *η*^2 ^= 0.1; High speed: *F*(1,35) = 5.05, *p *= 0.015, *η*^2 ^= 0.13). This finding indicates that under each target speed condition SPEM performance of the high schizotypy group was significant worse than that of the average schizotypy group. With respect to pupil diameter, ANOVA with Group as independent factor and Target speed as repeated measurements factor did neither indicate any significant difference between groups nor any significant interaction between Group and Target speed (Table 1).

**Table 1:** Means (M) ± Standard Deviations (SD) of pupil diameter for low, medium or high target speed and ANOVA test results in groups scoring average or high on SSQ-General Schizotypy

	Schizotypy				
	Average	High	*F (1,35)*	*p*	*η*^2^

Low speed	5.55 ± 0.97	5.39 ± 0.80	0.31	0.58	0.009

Medium speed	5.48 ± 0.96	5.27 ± 0.84	0.49	0.49	0.014

High speed	5.50 ± 0.96	5.26 ± 0.90	0.61	0.44	0.017

## Discussion

In this study we examined the relationship between subjects' scores on the *Schizotypic Syndrome Questionnaire *(SSQ) [48], pupil size and quality of smooth pursuit eye movements (SPEM), one of the most dependable biological markers of the genetic liability for schizophrenia [61]. In addition, to examine the effect of speed of the target on this relationship, subjects were instructed to follow a horizontally moving target under three conditions of speed. To assess deviant eye tracking, we used a global measure, the root mean square error score (RMSE) for eye vs. target position. We expected that higher scores on the (general factor of the) SSQ would be associated with more apparent SPEM dysfunction. We had no specific expectations on the relationship between target speed and magnitude of SPEM dysfunction. Finally, we examined whether there exists a relationship between General Schizotypy and baseline pupil diameter.

As a sampling frequency of 50 Hz is not optimal for measuring fast eye movements, the low temporal resolution used in the present study is a limiting factor for data interpretation. Therefore, conclusions drawn in the present study must be regarded with some caution.

In line with our expectations, we found more SPEM dysfunction in subjects scoring high on the general SSQ factor. As this result appeared valid for all target speed conditions, we may conclude that SPEM dysfunction in high General Schizotypy subjects is irrespective of the movement speed of the target. To interpret our findings correctly, it is important to note that we compared SPEMs of subjects scoring above 1 SD with subjects scoring between -1 and +1 SD on schizotypy, whereas other, similar studies made use of subjects scoring above 2 SD and between -0.5 and +0.5 SD [28-30]. Hence, it seems plausible to assume that the differences in SPEM dysfunction would even have been more pronounced if we had used these latter criteria. With respect to baseline pupil diameter no differences were found between the high and average scoring groups on General Schizotypy. These results are in agreement with what we expected.

As a measure for schizotypy we choose the SSQ concept of General Schizotypy. based on the confirmation of a model in subjects of the general population [43] and first-episode schizophrenic patients [50]. The 12 symptom scales of the SSQ constituted a network of causal relationships validly describing the process of schizophrenic prodromal unfolding and decompensation. As these scales were found to give rise to three correlated factors, the general factor of the SSQ was believed to present a dependable picture of the 'true' nature of schizotypy. Furthermore, this choice was based on indications that, in addition to the well-known relationship between negative symptoms of SPD or schizophrenia and SPEM dysfunction, also positive symptoms may relate to SPEM impairments. From a behavioural-genetic perspective these results are understandable as it is a generally accepted that the genetic predisposition to schizophrenia expresses itself predominantly in negative symptoms [31-36], although weaker relationships with that vulnerability factor have also been reported for positive symptoms [33]. There are even studies that indicate a primary association with positive symptoms of SPD [37,38]. As the SSQ factor General Schizotypy mainly pertains to negative symptoms, but also includes positive aspects [48], this instrument was considered to be quite suitable for use in the present study.

Although some researchers have suggested that the global quantitative RMSE may be more sensitive to SPEM dysfunction than precise quantitative parameters as pursuit gain or frequency of different saccade types [45], global and specific measures may in fact be complementary [62]. Several studies indicate that the underlying mechanism in SPEM dysfunction is the incidence of intrusive anticipatory saccades, reflecting a lack of inhibition in the system controlling saccades and eye fixations [63]. Indeed, accurate SPEM performance requires the subject to activate neural systems involved in smooth pursuit tracking, while simultaneously suppressing the activity of neurons responsible for saccadic movements that would move the eye ahead of the target. As the failure to suppress saccadic anticipation of target motion during smooth pursuit may be an aspect of SPEM dysfunction related to presumed risk for schizophrenia [64], it may well be true that the SPEM dysfunction we found in our subjects with elevated scores for General Schizotypy may be partly due to this inability to suppress saccadic anticipation, possibly mediated by the prefrontal cortex. Indeed, it has been argued that the frontal cortex may have a central role in the coordination and synchronization of effective SPEM performance, and attempts to characterize the basis of SPEM dysfunction in schizophrenia have emphasized the role of the frontal lobe in deviant SPEM performance [65,66]. However, as the anterior and posterior cingulate and subcortical systems such as basal ganglia, thalamus, and cerebellum have also been found to be involved in the smooth pursuit network [67], SPEM dysfunction in schizotypic subjects may be related to impairments in a diversity of brain areas.

## Conclusion

By investigating the General Schizotypy factor of the SSQ, the present study provides additional evidence that SPEM dysfunction is associated with schizotypic symptoms and is already observable in general population subjects scoring high on that factor. We further conclude that SPEM dysfunction may be used as an early diagnostic sign of schizophrenia.

## Competing interests

The authors declare that they have no competing interests.

## Authors' contributions

DvK conceived the idea and recruited the subjects, JBD performed the oculometric assessment, DvK and JBD analyzed the data, and DvK and JBD drafted the manuscript and approved the final paper.

## Pre-publication history

The pre-publication history for this paper can be accessed here:


